# Winnerless competition in clustered balanced networks: inhibitory assemblies do the trick

**DOI:** 10.1007/s00422-017-0737-7

**Published:** 2017-10-26

**Authors:** Thomas Rost, Moritz Deger, Martin P. Nawrot

**Affiliations:** Computational Systems Neuroscience, Institute for Zoology, Faculty of Mathematics and Natural Sciences, University of Cologne, Cologne, Germany

**Keywords:** Cortical variability, Attractor networks, Mean field theory, Binary networks, Multistability

## Abstract

Balanced networks are a frequently employed basic model for neuronal networks in the mammalian neocortex. Large numbers of excitatory and inhibitory neurons are recurrently connected so that the numerous positive and negative inputs that each neuron receives cancel out on average. Neuronal firing is therefore driven by fluctuations in the input and resembles the irregular and asynchronous activity observed in cortical in vivo data. Recently, the balanced network model has been extended to accommodate clusters of strongly interconnected excitatory neurons in order to explain persistent activity in working memory-related tasks. This clustered topology introduces multistability and winnerless competition between attractors and can capture the high trial-to-trial variability and its reduction during stimulation that has been found experimentally. In this prospect article, we review the mean field description of balanced networks of binary neurons and apply the theory to clustered networks. We show that the stable fixed points of networks with clustered excitatory connectivity tend quickly towards firing rate saturation, which is generally inconsistent with experimental data. To remedy this shortcoming, we then present a novel perspective on networks with locally balanced clusters of both excitatory and inhibitory neuron populations. This approach allows for true multistability and moderate firing rates in activated clusters over a wide range of parameters. Our findings are supported by mean field theory and numerical network simulations. Finally, we discuss possible applications of the concept of joint excitatory and inhibitory clustering in future cortical network modelling studies.

## Introduction

Neural responses in the mammalian neocortex are notoriously variable. Even when identical sensory stimuli are provided and animal behaviour is consistent across repetitions of experimental tasks, the neuronal responses look very different each time. This variability is found on a wide range of temporal and spatial scales (see e.g. Dinstein et al. 2015). To this day, it remains a matter of discussion how the brain can cope with this variability or whether it might even be an essential part of neural computation (e.g. Arieli et al. 1996; Masquelier 2013; Renart and Machens 2014).

Classically, neural variability has been interpreted as noise. In this view, there is a *signal* in the neural activity which is buried under some *noise*. By averaging over trials aligned to some experimental task, the noise is cancelled out and the *trial averaged firing rate* emerges as the signal. An obvious problem of this view is that trials are a construct imposed by the experimenter which the animal is likely unaware of. In contrast, animal behaviour works fine on a single trial basis where averaged signals are unavailable. It has been argued that averages could be taken instantaneously over populations of neurons coding for the same entity. If the *noise* carried by the individual neurons is uncorrelated, it can be eliminated in that way (Shadlen and Newsome 1998).

A potential source of the noise could be the thermodynamic or quantum mechanical randomness inherent in external stimulus modalities (Faisal et al. 2008). But then averaging should tend to reduce the amount of noise with increasing distance from the sensory periphery. Instead the opposite has been observed: cortical firing becomes increasingly variable in higher brain areas (Kara et al. 2000) being particularly high in motor cortex and again decreasing in motor periphery (Prut and Perlmutter 2003).

In earlier years, researchers attempted to explain neural variability using stochastic elements in detailed single neuronal models (e.g. Stein 1967). Later, it was shown that single neurons can precisely reproduce the same spike trains when repeatedly injected with identical current traces which resemble their integrated natural inputs (Mainen and Sejnowski 1995). Likewise, dendritic integration and synaptic variability cannot account for the large trial-to-trial variability in vivo (Nawrot et al. 2009; Boucsein et al. 2011). This in vitro observation of highly reliable signal integration and output firing patterns in single neurons raises the question how such seemingly chaotic activity can be induced in networks of deterministic units.

In neocortex, individual neurons receive large numbers of excitatory and inhibitory synaptic inputs from the surrounding network (e.g. Braitenberg and Schüz 1991; Larkman 1991; Destexhe et al. 2003). It has been shown that in network models of randomly connected excitatory and inhibitory neurons a condition exists in which these neurons fire in a chaotic manner at low firing rates. This condition was termed the *Balanced State* and occurs if excitation and inhibition to each cell cancel each other on average so that spike emission is triggered by fluctuations in the input current rather than by elevation of the mean input current (van Vreeswijk and Sompolinsky 1996, 1998; Brunel 2000). Using networks of binary units, van Vreeswijk and Sompolinsky (1996) showed that this dynamic equilibrium occurs without much fine tuning of the parameters if a few conditions are met. Few years later, now for networks of spiking (LIFs), Brunel (2000) characterised different types of firing activity in networks of LIFs as a function of the strength of an external drive to the network and the relative strength of excitation and inhibition. These balanced networks, however, could not yet explain the full extent of experimentally observed neuronal variability.

A recent series of studies (Deco and Hugues 2012; Litwin-Kumar and Doiron 2012; Doiron and Litwin-Kumar 2014; Mazzucato et al. 2015) have shown that competition between attractors can induce rate variation in balanced networks. Attractors were introduced into the networks by defining sub-populations or *clusters* in the excitatory populations of the networks and by increasing the synaptic efficacies (or weights) between units inside clusters relative to those to the remaining units. If the ratio of intra-cluster weights to inter-cluster weights is low, no change in the dynamics occurs. If the ratio is high, the attractors become *deep*, resulting in winner-take-all dynamics where one cluster has a high firing rate and suppresses the activity in the other assemblies (Lagzi et al. 2015). In the more interesting intermediate range of intra-cluster weights, the variance in the population firing rates causes the networks to *switch* between different states, each defined by a specific set of *active* clusters with higher firing rates (winnerless competition). This results in a scenario where individual units exhibit multistability in their firing rates and as a result introduce variance in firing rates that increases the trial-to-trial variability to levels that match those observed in vivo. In addition, selective stimulation of subsets of clusters causes certain attractors to become more stable, which in turn quenches the switching-dynamics, a mechanism that has been proposed as a potential model for working memory and perceptual bistability (Amit and Brunel 1997; Renart et al. 2007)

One problem with this family of models is that the active clusters tend to have firing rates close to saturation, where spike trains become very regular (clock-like). Also, the range of synaptic strength ratios in which state switching can occur is quite narrow.

In the present study, we analyse the dynamics of cluster competition and investigate possible improvements to the model. We first recapitulate the conditions of the balanced state using a binary neuron model for which an extensive mean field theory has been described (van Vreeswijk and Sompolinsky 1998; Renart et al. 2010). We then use the mean field approach to analyse the attractors of clustered networks and show that the introduction of inhibitory clustering can moderate the firing rates of the active clusters and facilitate winnerless competition among clusters.

## Balanced networks of binary neurons

We consider networks of $N_{E}$ excitatory and $N_{I}$ inhibitory binary units with asynchronous updates. Unless stated otherwise, a total of $N_{E}=4000$ and $N_{I}=1000$ units are used for numerical simulations. The ratio $N_{E}=4N_{I}$ is commonly used in cortical network simulation studies (e.g. Brunel 2000) and in accordance with anatomical statistics (Braitenberg and Schüz 1991). In each simulation step *t*, one unit is randomly chosen and its state $\sigma \in 0,1$ is updated according to the rule

1 $$\begin{aligned} \sigma _{i}(t+1) = {\Theta }\left( \sum _{j=1}^{N} J_{ij} \sigma _{j}(t) -\theta _{i} + J_{iX}m_{X}\right) , \end{aligned}$$

where ${\Theta }$ is the Heaviside step function, $J_{ij}$ is the synaptic weight between pre-synaptic unit *j* and postsynaptic unit *i*, $\theta _{i}$ is the threshold, and $m_{X}$ is the rate of some external drive to the unit which is modelled as a constant (rather than a spike source) weighted by $J_{iX}$. If a unit is updated to the *up*-state ($\sigma = 1$), it remains in that state until the next update. Hence, we may say that the network has an integration time scale $\tau$ equal to the average time between updates. Since time is not explicitly modelled, we assign a value of $\tau = 10$ ms similar to neuronal membrane time constants for illustration purposes only. Note that here $\tau$ is proportional to *N* due to the asynchronous update rule where the average time between consecutive updates of a specific unit depends on the total number of neurons. The update rule results in exponentially and independently distributed intervals between updates. van Vreeswijk and Sompolinsky (1998) have shown that the asynchronous and irregular activity of the model is independent of this aspect. The resulting spike statistics (transition times from $\sigma =0$ to $\sigma =1$) can, however, not meaningfully be compared to those of physiological data.

The connection strengths $J^{ij}_{\alpha \beta }$ from unit *j* in population $\beta$ to unit *i* in population $\alpha$ ($\alpha ,\beta \in E,I$) are set to $J_{\alpha \beta }$ with probability $p_{\alpha \beta }$ and to zero otherwise. Uniform connection probabilities between 1 and 20% are commonly used in the literature (e.g. Brunel 2000; Renart et al. 2010; Ostojic 2014; Kriener et al. 2014; Litwin-Kumar and Doiron 2014). To allow a comparison to other clustered network studies, we here adopt the approach taken by Litwin-Kumar and Doiron (2012) and Mazzucato et al. (2015) and set the connection probability of excitatory to excitatory units to $p_{EE} = 0.2$ and all those involving the inhibitory population to $p_{EI}=p_{IE}=p_{II}=0.5$. However, the principal results also hold for sparser connectivities.

Conditions for the values of the remaining model parameters arise from an analysis of the balanced state using the mean field description of the network dynamics described below.

### Mean field description of the balanced state

The following conditions for the balanced state and its stability arising from mean field considerations are adapted from van Vreeswijk and Sompolinsky (1998) and Renart et al. (2010). We guide the reader through some of the derivations as the references do not show them for the specific types of networks that we used here.

The balanced state requires spiking to be fluctuation driven, i.e. the mean inputs to each unit need to cancel while the variance has to be on the order of the spiking threshold. Since the number of inputs is proportional to the network size *N*, the synaptic weights have to be scaled with the square root of *N* to keep the variance constant for different network sizes. Hence, the synaptic strengths are scaled with network size as $J_{\alpha \beta } = j_{\alpha \beta }/\sqrt{N}$ where $j_{\alpha \beta }$ is a constant. Also, the number of input spikes required to reach the threshold needs to be small. We adopt the scaling used in van Vreeswijk and Sompolinsky (1998) so that $\sqrt{K}$ excitatory spikes arriving during one time constant suffice to elicit a postsynaptic one, where $K_{\alpha \beta } = p_{\alpha \beta }N_{\beta }$ is the average number of connections a unit in population $\alpha$ receives from population $\beta$. Inserting these assumptions into Eq. (1), this condition implies:

2 $$\begin{aligned} \sqrt{p_{\alpha E }N_{E}} J_{\alpha E} = \theta _{\alpha }. \end{aligned}$$

To achieve balance between excitation and inhibition, we need the excitatory and inhibitory inputs to each population to cancel. If excitatory and inhibitory population rates are equal, this means [again by Eq. (1)]:

3 $$\begin{aligned} 0= & {} N_{E} p_{EE} J_{EE} + \frac{1}{g} N_{I} p_{EI} J_{EI}, \end{aligned}$$

4 $$\begin{aligned} 0= & {} N_{E} p_{IE} J_{IE} + N_{I} p_{II} J_{II}. \end{aligned}$$

Here, we have introduced a factor *g* to control the relative strength of excitation and inhibition. When $g=1$, excitation and inhibition are equal, for $g>1$ inhibition dominates. This excess inhibition also allows for the accommodation of excitatory external inputs. Combining Eqs. (2) and (3), we can now compute the excitatory weights as:

5 $$\begin{aligned} j_{EE}= & {} \frac{\theta _{E}}{ \sqrt{p_{EE}n_{E}}} \end{aligned}$$

6 $$\begin{aligned} j_{EI}= & {} -g j_{EE}\frac{ p_{EE} n_{E}}{p_{EI}n_{I}} \end{aligned}$$

where $n_{E} = N/N_{E}$. Similarly, for the inhibitory population:

7 $$\begin{aligned} j_{IE}= & {} \frac{\theta _{I}}{ \sqrt{p_{IE}n_{E}}} \end{aligned}$$

8 $$\begin{aligned} j_{II}= & {} -j_{IE}\frac{ p_{IE} n_{E}}{p_{II}n_{I}}. \end{aligned}$$

The central assumption of the mean field approach is that for large *N*, the central limit theorem allows the treatment of the synaptic input to each unit as a Gaussian random variable (Renart et al. 2004). The dynamics of the mean population rates $m_{\alpha }(t)$ in networks of asynchronously updated binary units can then be described as (van Vreeswijk and Sompolinsky 1998; Renart et al. 2010):

9 $$\begin{aligned} \tau _{\alpha } \frac{\text {d}}{\text {d}t} m_{\alpha }(t) = -m_{\alpha }(t) + H\left( - \frac{\mu _{\alpha }(t)}{\sqrt{s^{2}_{\alpha }(t) }} \right) . \end{aligned}$$

Here, the population *activity*-rate $m_{\alpha }$ is defined as the average of the instantaneous states $\sigma \in [0,1]$ in population $\alpha$ [see Eq. (1)], $m_{\alpha }(t) = \langle \sigma _{\alpha } (t) \rangle$ and *H* is the complementary error function

10 $$\begin{aligned} H(z) = \frac{1}{\sqrt{2 \pi }} \int _{z}^{\infty } \text {d}x \mathrm{e}^{-\frac{x^{2}}{2}}. \end{aligned}$$

Note that $m_{\alpha }$ is not equivalent to the firing rate because spikes are only counted when units update their state from 0 to 1, while $\sigma$ remains at the value 1 until the next update. The population time constant $\tau _{\alpha }$ corresponds to the average time between successive updates of the states. $\mu _{\alpha }$ and $s^{2}_{\alpha }$ are the mean and variance of the input to population $\alpha$. The population average input is expressed as a function of the other population activities:

11 $$\begin{aligned} \mu _{\alpha }(t) = \sum _{\beta }\bar{J}_{\alpha \beta } m_{\beta }(t) +J_{\alpha X} m_{X} -\theta _{\alpha } \end{aligned}$$

where $\bar{J}_{\alpha \beta } = j_{\alpha \beta } p_{\alpha \beta }n_{\beta } \sqrt{N}$ is the average weight from population $\beta$ to $\alpha$.

The variance $s^{2}_{\alpha }$ arises from correlations between the inputs to a population as well as variance in the synaptic connectivity between units. Following van Vreeswijk and Sompolinsky (1996), we neglect correlations. The variance of the input is hence determined by the variance in the weights $\bar{J}^{(2)}_{\alpha \beta }$ (van Vreeswijk and Sompolinsky 1998; Renart et al. 2010), such that

12 $$\begin{aligned} s^{2}_{\alpha }(t) = \sum _{\beta } \bar{J}^{(2)}_{\alpha \beta }m_{\beta }(t). \end{aligned}$$

For constant weights, $\bar{J}^{(2)}_{\alpha \beta }$ is determined by the stochasticity in the connectivity. Since individual weights are either 0 or $J_{\alpha \beta }$ with probability $p_{\alpha \beta }$, the variance is that of a Bernoulli distribution and is computed as $\bar{J}^{(2)}_{\alpha \beta } = p_{\alpha \beta } \left( 1-p_{\alpha \beta } \right) j^{2}_{\alpha \beta } n_{\beta }$.

Equations (9) through (12) describe the transient dynamics of the system. When the external drive $m_X$ is constant, the activity rates eventually reach a steady state:

13 $$\begin{aligned} m_{\alpha } = H\left( - \frac{\mu _{\alpha }}{\sqrt{s^{2}_{\alpha } }} \right) . \end{aligned}$$

For large networks, the steady state rates can be found without explicitly solving Eq. (13). Since the number of synaptic inputs each unit receives is proportional to *N*, but the number of spikes required to make it fire is proportional to $\sqrt{N}$, the total magnitude excitation and inhibition arriving at each neuron is much larger than the firing threshold. Since the rates are required to be neither zero nor at the saturation limit, these large inputs have to cancel. This cancellation can happen only at precise values of the firing rates (Renart et al. 2010):

14 $$\begin{aligned} \bar{J}_{\alpha E} m_{E} + \bar{J}_{\alpha I} m_{I} + J_{\alpha X}m_{X} = 0. \end{aligned}$$

Rearranging Eq. (14), we can hence deduct the steady state population rates in the balanced state without solving Eq. (13):

15 $$\begin{aligned} m_{E}= & {} \frac{ \left( J_{EX} \bar{J}_{II} -J_{IX} \bar{J}_{EI}\right) }{\bar{J}_{EI}\bar{J}_{IE} - \bar{J}_{EE}\bar{J}_{II}} m_{X} \end{aligned}$$

16 $$\begin{aligned} m_{I}= & {} \frac{ \left( J_{EX} \bar{J}_{IE} -J_{IX} \bar{J}_{EE}\right) }{\bar{J}_{EE}\bar{J}_{II} - \bar{J}_{EI}\bar{J}_{IE}} m_{X} \end{aligned}$$

Using the definition of $\bar{J}_{\alpha \beta }$ and the expressions for the weights in Eqs. (5) through (8), we can express the balanced rates in terms of the network parameters and the strength of the external input:

17 $$\begin{aligned} m_{E}= & {} \frac{m_{x}}{\sqrt{N_{E}} \left( g-1\right) } \left( \frac{J_{EX} }{\theta _{E}\sqrt{p_{EE}}} - g \frac{ J_{IX}}{\theta _{I}\sqrt{p_{IE}}}\right) , \end{aligned}$$

18 $$\begin{aligned} m_{I}= & {} \frac{m_{x}}{\sqrt{N_{E}} \left( g-1\right) } \left( \frac{J_{EX} }{\theta _{E}\sqrt{p_{EE}}} - \frac{J_{IX} }{\theta _{I}\sqrt{p_{IE}}}\right) . \end{aligned}$$

For $m_E$ and $m_I$ to be positive and finite and assuming that $\theta _E = \theta _I$, we thus require either

19 $$\begin{aligned} g< 1 , \frac{J_{EX}}{J_{IX}} < g\frac{\sqrt{p_{EE}}}{\sqrt{p_{IE}} } \end{aligned}$$

or

20 $$\begin{aligned} g>1 , \frac{J_{EX}}{J_{IX}} > g\frac{\sqrt{p_{EE}}}{\sqrt{p_{IE}} }. \end{aligned}$$

If either Eq. (19) or (20) is satisfied, a fixed point with finite rates is ensured.

### Stability of the fixed points

Having deduced the steady state solutions or fixed points of the system, we now need to establish conditions for their stability. To assess the stability of fixed points of the network activity, we need to compute the partial derivatives of Eq. (9) with respect to $m_{\beta }$,

21 $$\begin{aligned} \begin{aligned}&\frac{\partial }{\partial m_{\beta }} \left( \frac{\text {d} m_\alpha }{\text {d}t}\right) = \\&\quad -\frac{1}{\tau _{\alpha }}\left( \frac{\partial m_\alpha }{\partial m_\beta } + H^{\prime } \left( - \frac{\mu _{\alpha }}{s_{\alpha } } \right) \frac{\bar{J}_{\alpha \beta } s_{\alpha } - \frac{1}{2}\mu _{\alpha } \bar{J}^{(2)}_{\alpha \beta } s^{-1}_{\alpha }}{s^{2}_{\alpha }}\right) \end{aligned} \end{aligned}$$

where

22 $$\begin{aligned} H^\prime (x) = - \frac{\mathrm{e}^{-\frac{x^{2}}{2}}}{\sqrt{2 \pi }} . \end{aligned}$$

If we denote the partial derivatives in Eq. (21) evaluated at a fixed point $\mathbf {m}_0$ as $\left. f_{\alpha \beta }\right| _{\mathbf {m}_{0}}$, we can write the stability matrix *S* as:

23 $$\begin{aligned} S = \left[ \begin{matrix} \left. f_{EE}\right| _{\mathbf {m}_{0}}&{}\,\left. f_{EI}\right| _{\mathbf {m}_{0}}\\ \left. f_{IE}\right| _{\mathbf {m}_{0}}&{}\,\left. f_{II}\right| _{\mathbf {m}_{0}} \end{matrix} \right] . \end{aligned}$$

For a locally stable fixed point of the firing rates, the real parts of both eigenvalues $\lambda _{1,2} = \frac{1}{2}\left( T_{S} \pm \sqrt{T^{2}_{S} -4 {\delta }_{S} }\right)$ of this matrix are required to be negative. Here, $T_{S}$ and ${\delta }_{S}$ are the trace and determinant of *S*. The eigenvalues are hence negative and real if the following conditions are fulfilled:

24 $$\begin{aligned} T_{S}< & {} 0 \end{aligned}$$

25 $$\begin{aligned} \left| T_{S}\right|> & {} \sqrt{T^{2}_{S} - 4\delta } \end{aligned}$$

26 $$\begin{aligned} T^{2}_{S}\ge & {} 4\delta . \end{aligned}$$

The partial derivatives (21) contain the population time constants $\tau _{\alpha }$. Since the values of $\tau _{\alpha }$ can be arbitrarily chosen, the stability of a fixed point depends only on their ratio (van Vreeswijk and Sompolinsky 1996). We can hence simplify the analysis by setting $\tau _{E}=1$. Defining $f^{\prime }_{\alpha \beta } =\left. f_{\alpha \beta }\right| _{\mathbf {m}_{0}} \tau _{\alpha }$, we can then make the dependence of the stability on the inhibitory time constant explicit and write:

27 $$\begin{aligned} T_{S}= & {} f^{\prime }_{EE} + f^{\prime }_{II}/\tau _{I} \end{aligned}$$

28 $$\begin{aligned} {\delta }_{S}= & {} \frac{1}{\tau _{I}}\left( f^{\prime }_{EE} f^{\prime }_{II}- f^{\prime }_{EI} f^{\prime }_{IE}\right) . \end{aligned}$$

Condition (24) is satisfied if $\tau _{I}< - \frac{f^{\prime }_{II}}{f^{\prime }_{EE}}$. Equation (25) is satisfied as long as $g>1$. Inserting (27) and (28) into (26) results in a quadratic equation in $\tau _{I}$. The system consequently has three bifurcations at the critical time constant ratios:

29 $$\begin{aligned} r_1= & {} A -\sqrt{A^2 - B^2} \end{aligned}$$

30 $$\begin{aligned} r_2= & {} -B \end{aligned}$$

31 $$\begin{aligned} r_3= & {} A + \sqrt{A^2 - B^2} \end{aligned}$$

with

32 $$\begin{aligned} A= & {} \frac{f^{\prime }_{EE}f^{\prime }_{II}-2f^{\prime }_{EI}f^{\prime }_{IE}}{(f^{\prime }_{EE})^2} \end{aligned}$$

33 $$\begin{aligned} B= & {} \frac{f^{\prime }_{II}}{f^{\prime }_{EE}} \end{aligned}$$

**Fig. 1:**
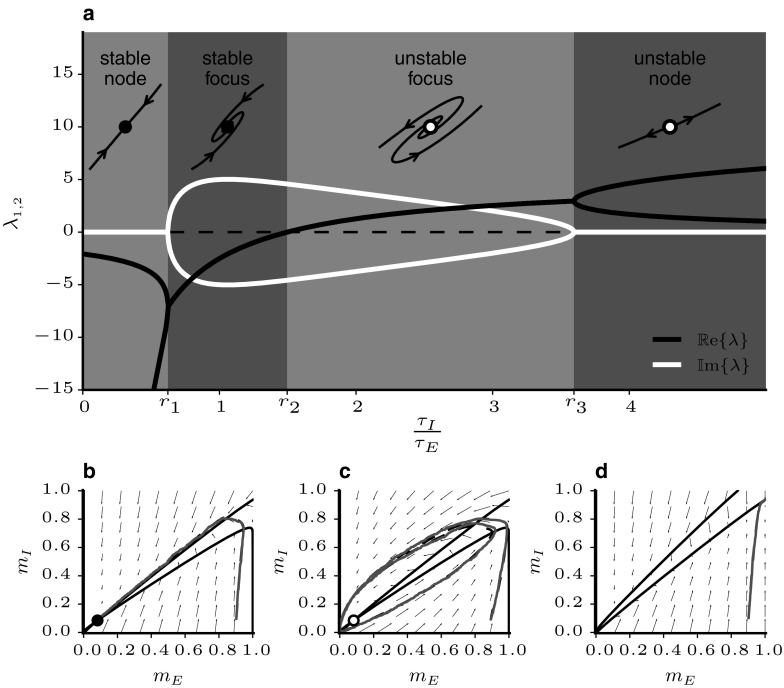
Illustration of stability and phase space analysis of binary balanced networks. **a** Dependence of eigenvalues of the stability matrix at the fixed point on the ratio of inhibitory and excitatory time constants. **b**–**d** Comparison of mean field description and simulations for networks with $N_{E}=4000$ illustrating various parameter settings. Dashed trajectories are mean field theory, grey traces are network simulations. Arrows represent derivatives of Eq. (9). Nullclines are drawn as solid black lines. Filled (empty) circles indicate stable (unstable) fixed points. **b** $g=1.2$, $\tau _{I}/\tau _{E} = 0.5$ **c** $g=1.2, \tau _{I}/\tau _{E} = 2$, **d** $g=0.8$. Remaining parameters are given in Table 1

The values of those ratios depend on the specific parameters, and their effect on the fixed point is illustrated in Fig. 1a. The system can show four qualitatively different types of behaviour as illustrated by the vertical grey stripes in the figure. For $\tau _{I}/\tau _{E} < r_1$, both eigenvalues are real and negative and the fixed point is a stable node, i.e. all trajectories in its vicinity converge directly towards it along the eigenvector corresponding to the largest eigenvalue. Above $r_1$, the eigenvalues become complex and the activity rates show damped oscillations towards the fixed points. When $\tau _{I}/\tau _{E} > r_2$, the fixed point becomes unstable and the firing rates escape towards an oscillatory limit cycle with large amplitude (see Fig. 1c). In simulations, different time constants for the populations are achieved by scaling the probability $P_{U\alpha }$ that a unit from population $\alpha$ is updated so that $P_{UE}/P_{UI} = \tau _I/\tau _E$.

The mean field theory described above shows good agreement with network simulation even for moderate values of *N*. Figure 1b–d shows some characteristic examples for the parameters given in Table 1. The activity rates for an excitatory and an inhibitory population are plotted against each other and arrows represent the derivatives at sample points in phase space. In panel b, the stability condition in Eq. (20) is met and $\tau _{I}/\tau _{E}<r_1$. The fixed point is hence a stable node and the simulated network rates behave as predicted by mean field theory and follow the flow field directly to the fixed point. In panel c, $\tau _{I}/\tau _{E}>r_2$ and the rates cycle through large amplitude oscillations. The match between mean field theory and simulation is also good when conditions (19) and (20) are violated (panel d).

Unless stated otherwise, we use the parameters summarised in Table 1 throughout.

## Clustered networks

Having established the conditions for the balanced state, we turn to introducing clustered connectivity in the excitatory population. Amit and Brunel (1997) modelled working memory and persistent activity in attractor networks using this approach, but did not consider variability dynamics. Their model consists of an unstructured background population and a number of attractor assemblies which are formed by increasing the weights between units belonging to the same assembly by a factor $J_+$, while across-cluster weights are decreased by a factor $J_-$ to maintain overall balance. A similar approach was taken by Mazzucato et al. (2015) and Deco and Hugues (2012) although the latter did not explicitly model the background or inhibitory populations. Litwin-Kumar and Doiron (2012) on the other hand increased the synaptic strength as well as the connection probabilities within clusters. In the following, we will analyse the fixed points of such networks using the mean field theory outlined in Sect. 2.1.

### Excitatory clusters

As we will see, an excitatory background population is not necessary for the clustering effects (see Fig. 4) and a simultaneous adjustment of synaptic strengths and connection probabilities complicates the analysis. We therefore choose in the present work to divide the excitatory population into *Q* equally sized clusters with uniform connection probabilities. Connections between units in the same cluster are multiplied by a factor $J_+ >1$, and to maintain a balance of weights, connections between units belonging to different clusters are multiplied by a factor

34 $$\begin{aligned} J_{-} = \frac{Q-J_{+}}{Q-1}. \end{aligned}$$

**Table 1:** Parameters used in the binary network simulations

Parameter	Value
$\theta$	1
$\tau _I$	$0.5 \tau _E$
$p_{EE}$	0.2
$p_{EI},p_{IE},p_{II}$	0.5
*g*	1.2
$J_{EX}$	$\sqrt{ p_{EE}N_{E}}$
$J_{IX}$	$0.8 \sqrt{ p_{EE}N_{E}}$
$m_{X}$	0.03

**Fig. 2:**
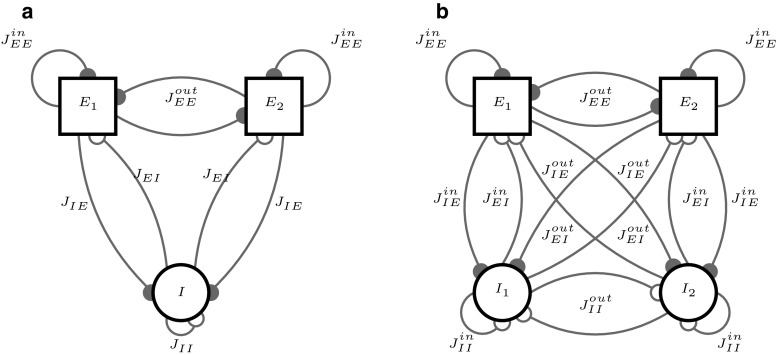
Architectures of networks with clustered connectivity. For clarity, only two clusters are used to show all connection types. **a** *EE*-clustering: two excitatory assemblies and a single unstructured inhibitory population. **b** *EI*-clustering: each excitatory cluster has an associated inhibitory population

Consequently, $J_{+}=1$ leads to homogeneous connectivity while at $J_{+}=Q$ the populations are completely decoupled. A schematic depiction of a network with $Q=2$ excitatory clusters and all occurring connections between populations is given in Fig. 2a.

**Fig. 3:**
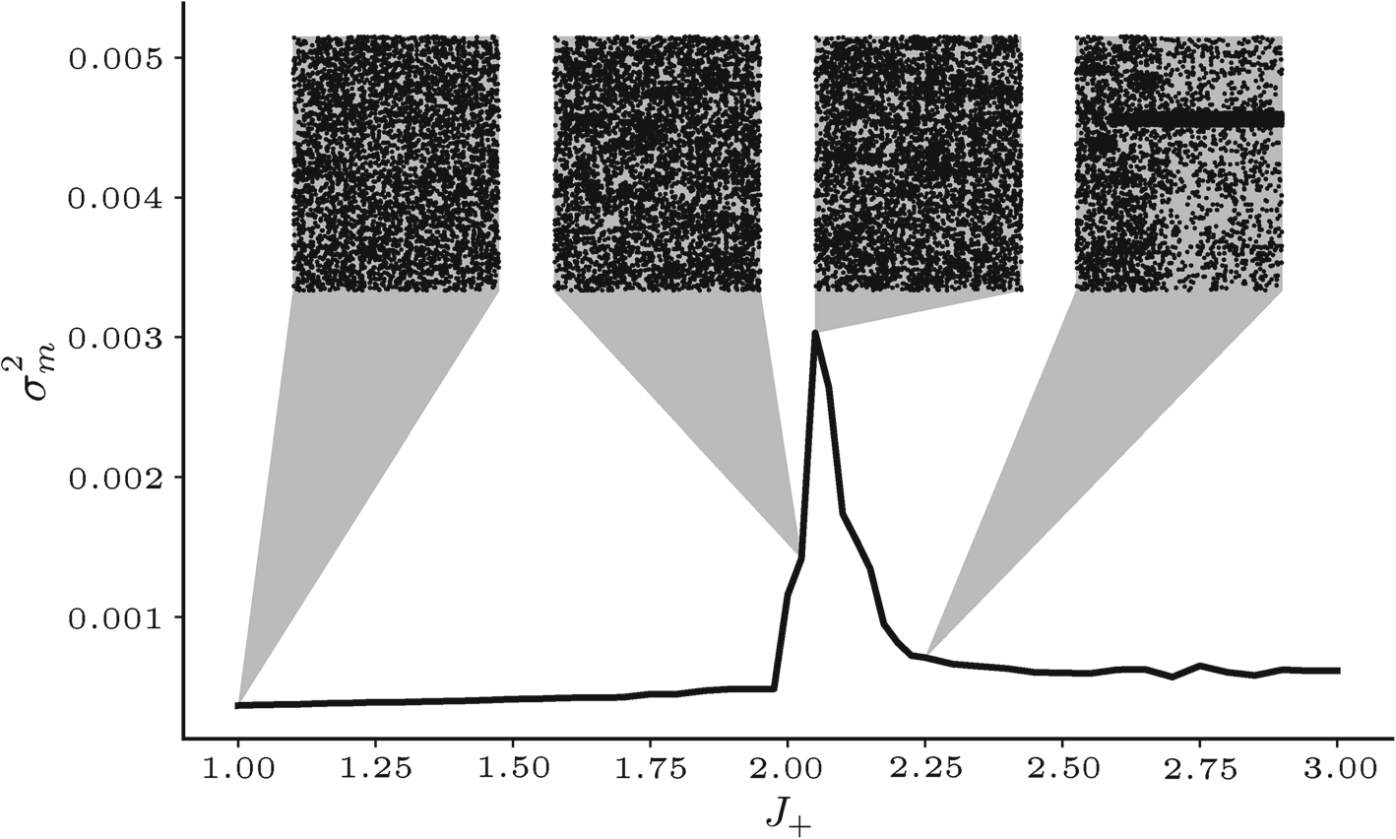
Activity rate variance in excitatory cluster networks. Average variance of the instantaneous mean cluster activity rates $\sigma ^2_m$ of 20 trials of 1000 ms duration for different values of the excitatory cluster strength $J_+$. The line shows the average over 20 network realisations. Insets show sample trials of spiking activity. Spikes were interpreted as the transition of a unit’s state $\sigma$ from 0 to 1

A common measure to quantify the effect of winnerless competition on the dynamics of networks of LIFs is the Fano Factor (FF) (Deco and Hugues 2012; Litwin-Kumar and Doiron 2012). The FF is defined as the variance of spike-counts over trials divided by the mean count (Shadlen and Newsome 1998; Nawrot et al. 2008; Nawrot 2010). Due to the random update process, the FF of spike trains from binary networks has no meaningful interpretation. When the activity rates saturate, i.e. when units fire each time they are updated, the FF is unity while for lower rates $\hbox {FF}>1$. Instead, to quantify the effect of clustering on the network dynamics we proceed as follows: We pool the activity of units in each cluster and calculate the mean instantaneous activity rates. We then compute the variance of these cluster rates over time. Finally, we average over clusters to obtain $\sigma ^{2}_m$ as an indirect measure. In unstructured networks, rate correlations between units are very low and hence there is little variance in the population-averaged activity rates. An increase in $\sigma ^{2}_m$ signifies correlated rate fluctuations of the units in a cluster. This means that individual clusters switch between activity states which is an indirect marker for winnerless competition.

Figure 3 shows the dependence of this variance on the cluster strength $J_+$ for networks with $Q=20$ clusters. At low values of $J_+$, the dynamics are not influenced by the clustered connectivity and $\sigma ^{2}_{m}$ does not change much compared to the unstructured case at $J_+=1$. Around $J_+\sim 2$, there is a sharp increase in rate variance. This is due to random activations of individual clusters in a winnerless competition regime that can be seen in the more structured looking raster plots in Fig. 3. After a sharp peak $\sigma ^2_m$ quickly drops again as clustering becomes so strong that clusters tend to remain active for increasingly long times, effectively producing winner-take-all dynamics (right-most raster plot). Note that the location of the peak with respect to $J_+$ depends on the number of clusters as well as on the size of the network (Litwin-Kumar and Doiron 2012).

To gain a better understanding of the underlying mechanisms, we employ the mean field approach to examine the stationary rate points of the clustered network. For this purpose, for each of the *Q* clusters as well as for the *I*-population an activity rate equation (Eq. (9)) is formed. Similar to the method described in Mazzucato et al. (2015), we then solve the resulting system of equations for the stationary states and check for stability as described in Sect. 2.1. It is worth noting that we consider only stable fixed points here. The mean field description is valid for very large *N*. For smaller networks, these attractors become *meta-stable* and switching between states occurs due to finite-size fluctuations in the population rates (Schwalger et al. 2017). This is different from the competition in Lotka–Volterra-like systems, where winnerless competition can be described by heteroclinic orbits between different saddle nodes (e.g. beim Graben and Hutt 2014).

Numerically solving such a multidimensional system of coupled differential equations requires that the initial guess for the solution is close to a fixed point. To sample the space of possible rate configurations, we therefore initialised the rates randomly between 0 and 1 and then integrated the system for a number of time steps before finding the exact fixed points using the Nelder–Mead simplex algorithm implemented in scipy (Jones et al. 2001). This process was repeated many times to ensure that most of the existing fixed points will be found. Note that the method can only find stable solutions of the system.

**Fig. 4:**
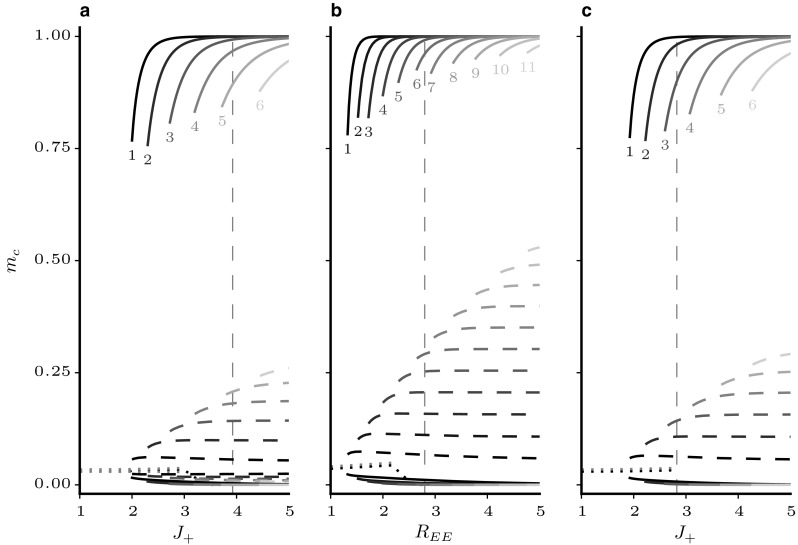
Stable state configurations in excitatory cluster networks. Sampled stable fixed points of the mean field equations for networks with 20 excitatory clusters versus the clustering parameter. Solid lines represent activity rates of *E* populations. For each solution, the number of clusters occupying a certain active state is written next to its onset. Dashed lines represent *I*-rates. The dotted lines show the case where all populations have the same rate. **a** Clustering by weight increase with 10% of the *E*-units as an unstructured background population as in Mazzucato et al. (2015), **b** clustering by increase in connection probability and synaptic strength as in Litwin-Kumar and Doiron (2012), **c** clustering by weight increase of the entire *E*-population as used in the present study. Vertical dashed lines correspond to the clustering strength where the homogeneous state is no longer stable

The resulting stable fixed points for different network structures are shown in Fig. 4. The solid lines with high rates indicate the rates of the clusters in the active state, while the lower solid lines represent the rates of the remaining populations. For a given parameter $J_+$, several solutions may exist, as indicated by the lines in different shades of grey. The numbers on the plots indicate how many populations occupy each state simultaneously. The remaining excitatory clusters occupy the down state. The activities of the inhibitory population are drawn as dashed lines, and the dotted line represents the homogeneous state where all excitatory populations fire at the same rate. To justify our choice of clustering method, the figure includes the attractor landscapes for networks with 10% of the units not belonging to any cluster (i.e. background population, panel a) as in Mazzucato et al. (2015), and for clustering of the connection probabilities as in Litwin-Kumar and Doiron (2012) (panel b). The clustering parameter $R_{EE}= p_\mathrm{in}/p_\mathrm{out}$ quantifies the ratio between connection probabilities within clusters to those across clusters. The results are not qualitatively different from networks without background population and cluster independent connection probabilities (panel c). As the cluster strength $J_+$ increases, more stable states occur with increasing numbers of simultaneously active clusters, resulting in multistability of the rates. At some critical value, the base-state in which all populations share the same low firing rate disappears. The common property of all three cases is that the *up*-states, i. e. the rates of the active clusters, show high activity rates and quickly approach the saturation value of 1 as the cluster parameter increases.

**Fig. 5:**
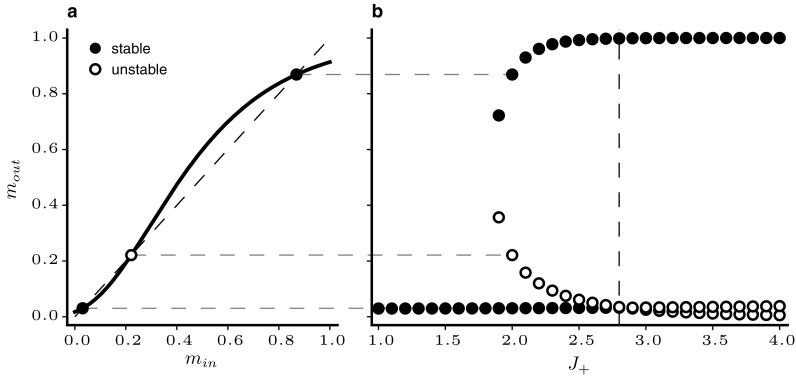
EFRs for states with a single active cluster in networks with excitatory clustering. Filled/empty circles represent stable/unstable fixed points. **a** Full EFR for $J_{+} = 2.0$, **b** fixed points of EFRs versus cluster strength $J_{+}$

It is evident in Fig. 4 that the state with the highest activity rate is always that with a single active cluster. That means that this rate forms an upper bound for the active cluster rates. We therefore carry on our analysis of the cluster dynamics by solving only for those cases. This is achieved by constraining $Q-1$ of the cluster populations to have equal rates. (Note that this could be extended to any number of active clusters.) Also, since the above-described method of random sampling of the activity space is costly and can yield only stable fixed points we employ a more systematic procedure for analysing the dynamics.

### Effective response functions

For single population models, the fixed points of neural activity can be found graphically by plotting the neurons’ gain function and the firing rates against the synaptic input and finding the intersections of the two lines (Gerstner et al. 2014). However, when the input to the gain function depends not only on one population rate, i.e. when there are several coupled differential equations, the graphical approach is no longer feasible. Mascaro and Amit (1999) describe an effective response function (EFR) approach for multipopulation models which puts one or more populations in focus while still incorporating the full dynamics of the remaining populations.

For a network model with *P* populations, the individual population rates can be expressed as functions of all the population rates in the network.

$$\begin{aligned} m_{1}= & {} {\Phi }_{1}\left( m_{1},m_{2} \ldots ,m_{P} \right) \\ m_{2}= & {} {\Phi }_{2}\left( m_{1},m_{2} \ldots ,m_{P} \right) \\ \vdots&\\ m_{P}= & {} {\Phi }_{P}\left( m_{1},m_{2} \ldots ,m_{P} \right) \end{aligned}$$

For the present case, ${\Phi }$ takes the form of Eq. (13). The EFR-approach (Mascaro and Amit 1999) works by treating the rate of a focus population as a parameter. That is, we fix $m_{1}=\bar{m}_{1}$ and solve the $P-1$ equations for the remaining rates.

$$\begin{aligned} m_{2}= & {} {\Phi }_{1}\left( \bar{m}_{1},m_{2} \ldots ,m_{P} \right) \\ \vdots&\\ m_{P}= & {} {\Phi }_{P}\left( \bar{m}_{1},m_{2} \ldots ,m_{P} \right) \end{aligned}$$

The solution $m^{\prime } \left( \bar{m}_{1}\right)$ to those equations will drive the rate of the focus population to a value $m_{1\mathrm{out}}$ given by

35 $$\begin{aligned} m_{1\mathrm{out}} = {\Phi _{1}}\left( \bar{m}_{1},m^{\prime } \left( \bar{m}_{1}\right) \right) = {\Phi _{eff}}\left( \bar{m}_{1}\right) . \end{aligned}$$

Mascaro and Amit (1999) called the resulting input–output relation for the focus population the EFR. When $m_{1\mathrm{out}}=\bar{m}_{1}$, i.e. when the EFR intersects the diagonal, $m_{1\mathrm{out}}$ is a fixed point of the system. If the slope of the EFR at the intersection is larger than unity, the fixed point is unstable. For slopes smaller than unity, the fixed point is stable for $m_{1\mathrm{out}}$ given $m^{\prime } \left( \bar{m}_{1}\right)$. Since this is a one-dimensional representation of potentially multidimensional systems and since the stability of fixed points depends additionally on the ratios of population time constants which are not captured by the EFR, those points are not generally globally stable for the whole system (Mascaro and Amit 1999). We therefore assess the stability of fixed points by examining the eigenvalues of the stability matrix.

**Fig. 6:**
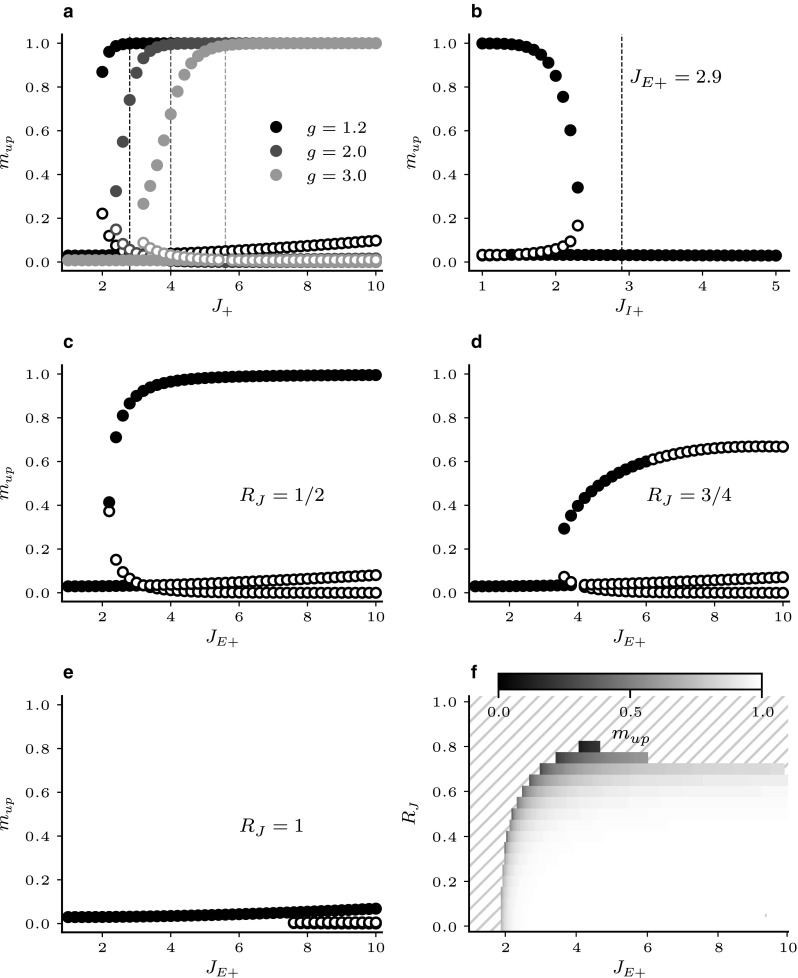
EFR fixed points for a single active population for networks with excitatory and varying degree of inhibitory clustering. Filled/empty circles represent stable/unstable fixed points. **a** $m_\mathrm{up}$ versus $J_{+}$ for different values of global inhibitory strength *g*. **b** $m_\mathrm{up}$ vs $J_{I+}$ with $J_{E+}$ held constant at the value where the homogeneous fixed point becomes unstable if $J_{I+}=1$. **c**–**e** Proportional increase of $J_{E+}$ and $J_{I+}$ for different strength ratios $R_J$. **f** Stable $m_\mathrm{up}$ as a function of $J_{E+}$ and $R_J$. Hatching indicates that no stable up-states are present

**Fig. 7:**
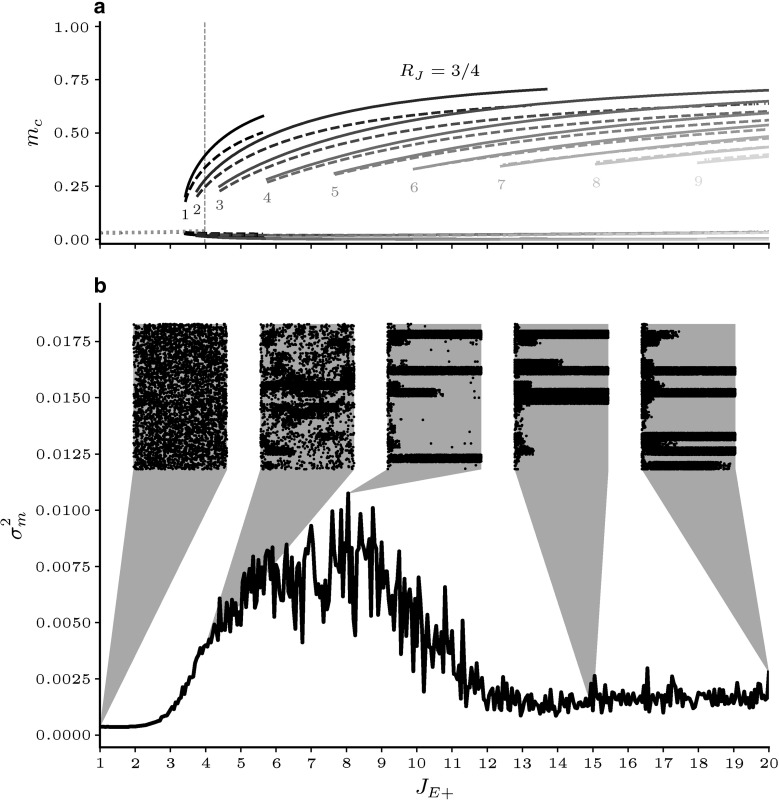
Stable state configurations and activity rate variance for *EI*-cluster networks. Network dynamics with $Q=20$ and $R_J = 3/4$ with excitatory cluster strength varied from $J_{E+}=1$ to full excitatory decoupling at $J_{E+}=Q$. **a** Stable rate fixed points of the unconstrained mean field equations of the system. **b** Instantaneous variance in mean cluster activity rates averaged over 20 network realisations

**Fig. 8:**
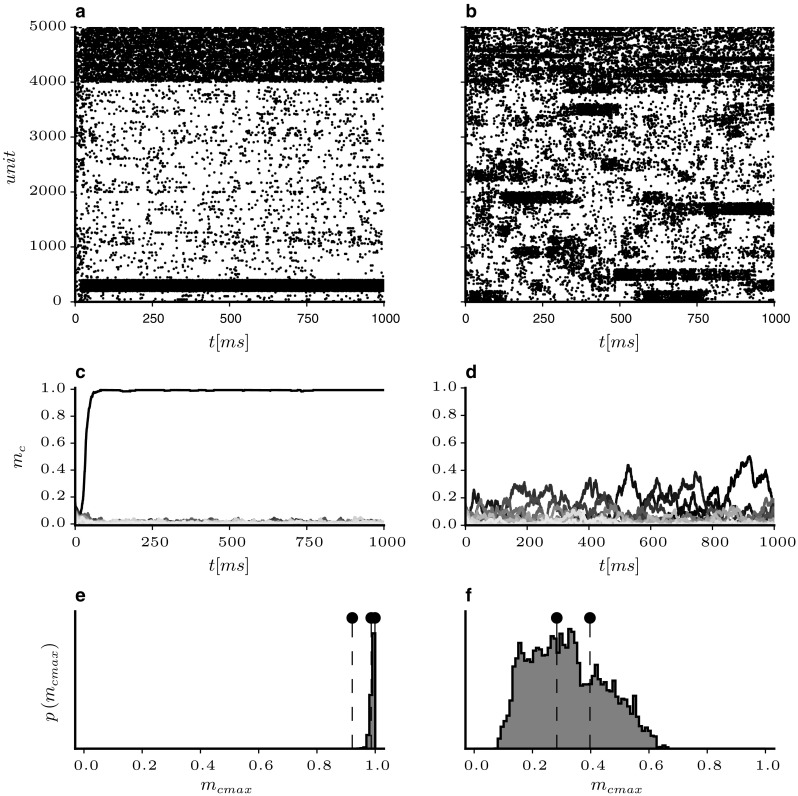
Comparison of *E* and *EI*-cluster simulations. Cluster dynamics with $J_{E+} = 2.9, R_J=0$ (left) and $J_{E+} = 4,R_J=3/4$ (right). **a**, **b** Sample raster plots of *E* and *I* spiking activity, **c**, **d** mean cluster activity rates $m_c$ for the trial shown above. Dashed lines show cluster rates smoothed with a Gaussian kernel with $\sigma =75$ ms. **e**, **f** Distributions of instantaneous maximum cluster activity rates $\mathrm{max} \left( m_{c}\right)$ for 100 random network realisations. Fixed points from mean field theory are indicated as dashed lines

Figure 5 illustrates the EFR for $Q=20$ clusters and a single unconstrained cluster (corresponding to the line labelled 1 in Fig. 4c). Panel a shows the full EFR for $J_{+} = 2.0$ where Eq. (35) has been evaluated on a dense grid for the whole range of activity rates. This representation reveals an additional unstable fixed point between the low rate attractor where all excitatory populations have the same firing rate and the *up*-state of the focus population. In panel b, the EFR has been calculated for different cluster strengths and only the fixed points of the system are plotted versus $J_{+}$. At $J_{+}=1$, the EFR is simply a flat line. That is, an increase in $m_\mathrm{in}$ has no significant effect on $m_\mathrm{out}$. As $J_{+}$ increases, the self-amplification of the focus population causes $m_\mathrm{out}$ to increase with $m_\mathrm{in}$ until the EFR touches the diagonal. At this point ($J_{+}\sim 1.8$), a stable *up*-state with an intermediate unstable fixed point emerges.

To switch between *up*-states of different populations, the variance in the population activities has to be sufficient to cross the unstable fixed point. It can be seen in panel b that this becomes increasingly less likely as the separation between fixed points widens with increasing $J_+$. The peak of $\sigma ^{2}_{m}$ in Fig. 3 occurs only in the narrow range where stable *up*-states exist at rates below the saturation limit. A potential solution to the problem of high firing rates could be to simply increase the strength of inhibition. Changing the relative inhibition parameter *g* has, however, not yielded qualitatively different attractor structures. In Fig. 6a, the plot from Fig. 5b has been reproduced for higher values of *g*. The dashed lines again represent the value of $J_{+}$ at which the homogeneous state becomes unstable. At these points, the active states are again approaching the saturation rate also for stronger global inhibition. Since inhibition is global, an increase in firing rate of a single population leads only to a small rise in inhibitory activity, so that the self-excitation of the active cluster is not balanced and the rate saturates at the upper limit.

We, therefore, hypothesise that in order to reduce the firing rates of active clusters it is necessary that inhibition is also cluster specific so that a rate increase in an excitatory cluster is *balanced* by a corresponding inhibitory population. This idea will be explored in the following section.

### Excitatory–inhibitory clusters

We have seen in the previous section that excitatory clusters in networks with global inhibition lead to rate saturation in active clusters which impedes state switching because the active and inactive cluster states are far apart (Fig. 5b). We will now show how this problem can be overcome by introducing structure in the inhibitory connections as well.

Excitatory–inhibitory (*EI*) clusters have previously been described in the context of persistent activity (Aviel et al. 2004; Renart et al. 2007). Litwin-Kumar and Doiron (2012) briefly describe how clustering the inhibitory units leads to stimulation-induced variability reduction in the inhibitory units. We will now investigate the concept of inhibitory clustering in detail, with specific focus on its effect on activity rates.

In *EI*-clustered networks, we require that an *E*-population selectively excites its corresponding *I* population which in turn selectively inhibits the *E* units. It is therefore necessary to close the loop and cluster both the *EI* and *IE* synapses. Like the *E*-population, the inhibitory units are equally divided into *Q* clusters, resulting in a total of 2*Q* populations. We rename the clustering factor for the excitatory population as $J_{E+}$. For simplicity, all connections involving the inhibitory population are lumped into a single cluster parameter $J_{I+}$. Balance is again maintained by rescaling across-cluster connections according to Eq. (34) so that the average row sum remains constant in each quadrant of the connectivity matrix. An overview over the possible connections is given in Fig. 2b for $Q=2$, where within/across-cluster connections are denoted by the superscript in/out. So, we have $J^\mathrm{in}_{EE} = J_{E+} J_{EE}, J^\mathrm{out}_{EE} = J_{E-} J_{EE}$ and $J^\mathrm{in}_{\alpha \beta } = J_{I+} J_{\alpha \beta }, J^\mathrm{out}_{\alpha \beta } = J_{I-} J_{\alpha \beta }$ for $\alpha \beta \in (EI,IE,II)$.

Since we have seen before that the highest *up*-state rates ($m_\mathrm{up}$) are always reached by a single active cluster, we again constrain the population equations, so the $Q-1$ excitatory as well as their corresponding inhibitory populations have the same rate, resulting in a total of four distinct equations to solve. To examine the effect of inhibitory clustering, we start by keeping $J_{E+}$ fixed at 2.9 (the first point in Fig. 5 where the homogeneous state is unstable) and then increase $J_{I+}$. The resulting fixed points are shown in Fig. 6a.

For low values of $J_{I+}$, $m_\mathrm{up}$ is again at the saturation limit. As the inhibitory cluster strength increases, however, the active cluster rates decrease and the stable homogeneous state reappears. When $J_{I+}$ increases further, a bifurcation occurs and the active cluster state no longer exists. This can be understood intuitively. An increase in $J_{I+}$ strengthens the coupling between the active cluster and its corresponding inhibitory population. When the coupling becomes too strong this selective inhibition prevents the focus population from attaining higher firing rates. It can therefore be concluded that low firing rates in active clusters can be obtained if the inhibitory cluster strength is present but smaller than the excitatory parameter.

Having established that the inhibitory clustering needs to be weaker than that of the *E* population, we introduce a proportionality factor $R_J$, so that

36 $$\begin{aligned} J_{I+} = 1 + R_J \left( J_{E+}-1\right) . \end{aligned}$$

That is, when $R_J = 0$, the inhibitory connections are un-clustered and for $R_J=1$ we have $J_{E+} = J_{I+}$. Having defined a relationship between the excitatory and inhibitory cluster parameters, we can now examine the fixed point landscape for different values of $R_J$. This is shown in Fig. 6c–e. Here, $J_{E+}$ is varied over a wide range. It can be seen in the sequence of plots that increasing $R_J$ has three effects. Firstly, it moves the appearance of *up*-states to higher values of $J_{E+}$. Secondly, it causes the *up*-states to become unstable when both $R_J$ and $J_{E+}$ are high. Finally, an increase in $R_J$ leads to a gradual decrease in the maximum rates reached by the active cluster. For $R_J$ close to one, the regime where stable *up*-states exists becomes increasingly narrow and when $J_{E+}=J_{I+}$ the active cluster states vanish (Fig. 6e). Figure 6f shows only the stable up-states with a single active cluster for a wide range of $R_J$ and $J_{E+}$. If configurations with more than one active cluster are considered, the range in which stable solutions are found increases (not shown).

We have hence shown that increasing excitatory and inhibitory cluster strength proportionally can yield the desired effect of preventing the active cluster rates from saturating and consequently reducing the gap between *up* and *down* states which should in turn facilitate spontaneous switching between active clusters.

Figure 7 illustrates that this is indeed the case. Panel a shows sampled fixed points of a network with 20 clusters and $R_J=3/4$ in analogy to the illustration in Fig. 4c (no inhibitory clustering). This time we have increased $J_{E+}$ all the way to *Q*, at which point the inter-cluster connections of the excitatory populations vanish. It can be seen that over the whole range of cluster strengths, the maximum rates do not exceed 0.7. Also, even when the excitatory clusters are fully decoupled, there are still multiple different configurations of active clusters. The dashed lines below the *up*-states represent the inhibitory counterparts to the active clusters. This selective increase in inhibition is what prevents the active states from saturating.

Panel b shows the corresponding rate variance plot with sample raster plots. Compared to the equivalent plot for excitatory clustering only in Fig. 3, the peak of $\sigma ^{2}_m$ has shifted to higher clustering strengths, while its amplitude and width have at the same time increased substantially. The interesting range for neural computation is likely to lie in the rising branch of the $\sigma ^{2}_m$ curve where winnerless competition occurs but for illustration purposes, the whole range is shown. At $J_{E+}=4$, the point where the homogeneous state becomes unstable, the activity cycles through the clusters with moderate firing rates as desired. For higher cluster strengths, the activities of the *down*-state clusters become increasingly suppressed and the active clusters remain in the *up*-states for increasingly long times. The sample raster plots show, however, that switching between states still occurs even when the excitatory clusters are fully decoupled at $J_{E+}=20$.

In Fig. 8, two cases with and without inhibitory clustering are compared in more detail. Since inhibitory clustering shifts the onset of cluster dynamics to higher values of $J_{E+}$, we compare the dynamics at the point where the homogeneous state has just become unstable for each architecture. The left panels of the figure show the case where $J_{E+} = 2.9$ and $R_J=0$. On the right-hand side (RHS), $R_J$ was 3 / 4 and $J_{E+}=4$.

Since the homogeneous state is unstable for the parameters chosen, the activity quickly moves from the random initial state to an active cluster in both cases. In the raster plot in panel a, it is evident that the network remains in that state for the remainder of the simulation period for *EE*-only clustering. The firing rate plot in panel c confirms that the active cluster immediately goes into rate saturation. Since this increases the rate of the inhibitory population, all other *E*-populations experience a reduction in rate, which further widens the gap between the high and low cluster states. In the *EI*-clustered network on the other hand (panel b), the activity cycles through different attractors and multiple clusters can be active simultaneously. It can be seen that the inhibitory clusters closely follow the rate excursions of their excitatory counterparts. Although all cluster have equal sizes and the weights of the same type (i.e. within or across populations) are all identical, the switching between active clusters seems to occur at random. As is made evident from the individual cluster activity rates depicted in panel d, different clusters fall into active states at different times with moderate rates.

The firing rates for the *EI*-clustered network also seem to follow the predictions obtained from the mean field model. To illustrate this, we have plotted the distributions of instantaneous maximum cluster activity rates for 100 repeated network simulations in the bottom panels of Fig. 8. The dashed lines and dots represent the stable *up*-states predicted by the mean field model. For the *E*-only cluster model, the theory predicts three different stable configurations (one, two and three active clusters, respectively). However, in 100 separate simulation runs almost exclusively the state with a single saturated active cluster was reached (panel e). For the *EI*-case, the theory yielded two stable configurations. The maximum rates obtained from network simulations had a wider distribution, but the shape coincides with the theoretical fixed points. The configuration with two active clusters at lower rate seems to occur more frequently than the higher rates of individual clusters. Maximum rates were higher than the stable points predicted by the model. Note, however, that the probability of finding activity rates higher than $\sim 0.7$ was zero, i.e. rate saturation occurred in none of our simulations.

## Discussion

### Clusters, winnerless competition and inhibition

Various configurations of excitatory cluster architectures have been examined with respect to winnerless competition (Deco and Hugues 2012; Litwin-Kumar and Doiron 2012; Doiron and Litwin-Kumar 2014; Mazzucato et al. 2015) and some previous reports exist of attractor dynamics with inhibitory assemblies. Schaub et al. (2015) demonstrated winnerless competition between inhibitory and excitatory clusters, while Litwin-Kumar and Doiron (2012) showed that the stimulation-induced reduction in spike count variance can also be found in inhibitory assemblies. However, neither of these studies implemented specific and reciprocal pairwise interactions between excitatory and inhibitory clusters in both directions which avoid rate saturation. Renart et al. (2007) used recurrently coupled excitatory–inhibitory clusters and showed that persistent activity at low rates with irregular firing is possible. They did, however, not vary the cluster strengths for excitation and inhibition separately and therefore required fine tuning of the parameters to obtain bistable configurations. Instead, the *EI*-cluster configuration presented here allows for winnerless competition over a wide range of cluster strengths (cf. Fig. 7).

### Plausibility of cluster-specific inhibition

Having established the dynamical advantages of *EI*-clustering, the question arises whether such an architecture can be justified based on anatomical, morphological and physiological evidence. For excitatory neurons, local connections are much more likely than longer projections [e.g. Schnepel et al. (2015), for review see Boucsein et al. (2011)] and *small world* structures have been reported on many spatial scales (Sporns and Zwi 2004). Bidirectional connections as well as clustered three-neuron patterns are much more frequent than would be expected in a random network (Song et al. 2005). Such motives would also tend to have stronger connections.

In network simulations, the units are commonly only divided into excitatory and inhibitory cells, while anatomical studies have identified various different types inhibitory interneurons (e.g. Markram et al. 2004; Harris and Shepherd 2015) and a single excitatory neuron may make connections with several interneuron types (Markram et al. 1998). An intermediate physiological level of detail is the distinction between fast-spiking (FS) and non-FS inhibitory interneurons. In addition to their different spiking behaviour implied by their names, these two cell types exhibit different connection schemes which imply functional differences. Fast-spiking cells are mainly locally connected, while non-FS neurons make translaminar connections (Dantzker and Callaway 2000; Levy and Reyes 2012; Kätzel et al. 2011; Otsuka and Kawaguchi 2009). On a finer scale, most connections between excitatory and FS-interneurons are reciprocal (Holmgren et al. 2003) with inhibitory postsynaptic currents being three times larger in reciprocal connections than for unidirectional ones (Yoshimura and Callaway 2005). With distance from an excitatory unit in a small volume, the connection probability with local inhibitory units varies only slightly, but the fraction of reciprocal connections decreases (Holmgren et al. 2003). Reciprocally connected cell pairs also share more common input than non-connected or unidirectionally connected pairs, while non-FS-cells share little common input, connect to excitatory units with lower probability and reciprocal connections are rare and not stronger than unidirectional ones (Yoshimura and Callaway 2005). Also, interneurons of different types are frequently connected with each other (Reyes et al. 1998) and inhibition can also be exerted bisynaptically so that excitatory axons excite inhibitory cells local to other populations (Binzegger et al. 2005).

It is hence safe to say that there is a lot of structure in the inhibitory cortical connectivity. The strong reciprocal and local inhibition of the FS-cells and the weaker longer range connections of the non-FS-interneurons could provide a substance for the type of inhibitory clustering we have proposed. Whether inhibition is less localised than excitation, as predicted by our model, cannot be conclusively answered at this time. The current physiological evidence certainly does not rule out the possibility.

The connectivity scheme presented here is of schematic nature. For excitatory connections, it has been shown that slow firing rate variations can also be achieved with overlapping clusters (Litwin-Kumar and Doiron 2012), mutually connected weight hub units with strong inward synapses (Setareh et al. 2017) or a range of other connectivity types (Doiron and Litwin-Kumar 2014). Although the exact implementation may vary, we believe that our proposal of local balancing of structured networks provides a very robust solution for metastability. A natural step for extending our work would be to introduce a spatial topology that maps to structural and functional local excitatory and inhibitory connectivities in cortical networks (see e.g. Rosenbaum et al. 2017). Local balance could then be achieved by strong short-range and weaker long-range inhibitory connections without explicitly assigning clusters in the inhibitory population.

## Conclusions and prospects

In this article, we have applied the mean field theory of networks of binary neurons to balanced networks with clustered sub-populations. We have shown that multistability with moderate firing rates can be achieved in balanced networks with joint excitatory and inhibitory clusters. This architecture allows for robust winnerless competition dynamics without rate saturation over a wide range of cluster strengths.

### Spike-train statistics in *EI*-networks

The binary neuron model does not meaningfully allow the analysis of spike-train statistics. It will hence be interesting to investigate the variability in *EI*-cluster networks of spiking LIFs. Networks with excitatory clusters capture the high spike count variance as measured by the *FF* (Deco and Hugues 2012; Litwin-Kumar and Doiron 2012) as well as its reduction during stimulus presentation which has been found in a range of cortical data sets (Rickert et al. 2009; Churchland et al. 2010). But the high firing rates in active (*E*-only) clusters lead to very regular firing. As shown by Renart et al. (2007), balancing excitatory and inhibitory clusters can lead to persistent activity with irregular inter-spike intervals. We therefore predict that the model presented here can conserve the irregular firing observed in cortical data during winnerless competition. The behaviour of count and interval statistics during external stimulus application remain a subject of further study. It has been suggested that the cellular mechanisms of spike frequency adaptation can further contribute to the experimentally observed variability dynamics (Farkhooi et al. 2011, 2013). In future modelling studies, cellular and network effects on the *FF* should be integrated.

### Increased robustness

Winnerless competition relies on the switching of activity between attractors which is due to fluctuations in the firing rates of individual clusters. In networks with excitatory clusters, switching will therefore only occur if clusters are small enough for the average rates to retain some variance. Further, inhomogeneities in cluster size can hamper competition so that larger clusters will tend to *win* once they switch to an active state. Because the firing rates of high and low states are closer together in *EI*-cluster networks, the model presented here alleviates both issues and will likely show more robust winnerless competition over a wider range of parameters and in the case of heterogeneous network parameters. In cortex, network conditions are constantly changing (Arieli et al. 1996; Schmidt et al. 2016). Robustness thus is likely to be a requirement for more realistic cortical network models.

### Computational role of winnerless competition

So far, we have discussed the dynamics of activity switching between clusters only from the perspective that it provides a mechanistic explanation for the high rate variance observed in cortex and its quenching by stimulation.

Traditionally, stable patterns or attractors in networks are used to model working memory (e.g. Hopfield 1982; Amit and Brunel 1997; Aviel et al. 2004). A network is *pushed* into a certain attractor by some external drive and maintains a certain configuration of firing rates which can be read out or *retrieved* at a later point. The difference between this scenario and the dynamics of winnerless competition is simply the *depth* of the attractors as characterised by the strength of clustering in the connectivity structure. This relationship lends itself to the interpretation that the stability of attractors is related to the probability of some variable encoded in their firing rate.

Mounting physiological indications exist for the hypothesis that some correlate of prior probability over previously observed states is encoded in the spontaneous activity in the neocortex. Berkes et al. (2011) report that the spontaneous activity in the visual cortex of ferrets before eye-opening is unstructured and becomes more and more similar to that evoked by visual stimuli during development. They consequently interpret the spontaneous activity as the prior probability of observing certain patterns and fit a Bayesian model to their data. This observation is well matched by the results of Kenet et al. (2003) and Luczak et al. (2009) who found that in auditory and somatosensory cortex of rats, spontaneous states resemble those evoked by sensory stimulation.

There is also more direct evidence that these priors are actually used during perception. Supèr et al. (2003) report that significant differences were detected in the firing rates in monkey visual cortex between trials where monkeys correctly reported the occurrence of a stimulus and those where they missed it. Similarly, Hesselmann et al. (2008) found that perceptual decisions can be predicted from ongoing activity in fMRI signals in humans 1.5 s prior to stimulus presentation in a face-or-vase task. In light of the theory presented here, it can be interpreted that the attractor that a network is currently in influences what decision is made.

Contrary to our fixed random network connectivity, structure is shaped by synaptic plasticity in vivo. A number of studies have recently been published, where clusters of excitatory units form in balanced networks through spike-time dependent plasticity (STDP) and selective stimulation (Ocker et al. 2015; Zenke et al. 2015; Litwin-Kumar and Doiron 2014). The resulting connectivities exhibit high rate variability in the spontaneous state. In a related study, binary networks with synchronous updates using STDP-inspired as well as homeostatic learning rules were shown to perform Bayesian-like inference in sequence-learning tasks (Lazar et al. 2009).

In all the above studies, some form of inhibitory plasticity was used as a homeostatic mechanism. Homeostasis seems to be generally required for networks with excitatory plasticity to prevent positive feedback loops (Zenke et al. 2013). For example Litwin-Kumar and Doiron (2014) use an inhibitory STDP rule for the *EI*-connections, i.e. the synapses that mediate inhibition from the inhibitory to the excitatory population to prevent winner-take-all dynamics. Our results predict that the *EI* as well as the *IE* connections are to some extent plastic to achieve the specificity required to obtain local balance in each assembly. It remains a subject of further study how such self-organisation can be achieved in simulations and whether it can be found in biological circuits.
